# Shunt Intervention for Possible Idiopathic Normal Pressure Hydrocephalus Improves Patient Outcomes: A Nationwide Hospital-Based Survey in Japan

**DOI:** 10.3389/fneur.2018.00421

**Published:** 2018-06-07

**Authors:** Madoka Nakajima, Masakazu Miyajima, Ikuko Ogino, Chihiro Akiba, Kaito Kawamura, Michiko Kurosawa, Nagato Kuriyama, Yoshiyuki Watanabe, Wakaba Fukushima, Etsuro Mori, Takeo Kato, Hidenori Sugano, Kostadin Karagiozov, Hajime Arai

**Affiliations:** ^1^Department of Neurosurgery, Faculty of Medicine, Juntendo University, Tokyo, Japan; ^2^Department of Epidemiology and Environmental Health, School of Medicine, Juntendo University, Tokyo, Japan; ^3^Department of Epidemiology for Community Health and Medicine, Graduate School of Medical Science, Kyoto Prefectural University of Medicine, Kyoto, Japan; ^4^Department of Public Health, Faculty of Medicine, Osaka City University, Osaka, Japan; ^5^Department of Psychiatry, Graduate School of Medicine, Osaka University, Osaka, Japan; ^6^Department of Neurology, Hematology, Metabolism, Endocrinology, and Diabetology, Faculty of Medicine, Yamagata University, Yamagata, Japan

**Keywords:** Alzheimer's disease, cerebrospinal fluid shunt, normal pressure hydrocephalus, prognosis, epidemiological survey

## Abstract

**Background and Purpose:** This study aimed to investigate the efficacy of cerebrospinal fluid shunt intervention for idiopathic normal pressure hydrocephalus (iNPH) using data from a nationwide epidemiological survey in Japan.

**Methods:** We conducted a cross-sectional study using data from a nationwide epidemiological survey performed in Japan. Propensity score matching was used to select 874 patients from 1,423 patients aged ≥60 years, who were diagnosed with iNPH based on clinical guidelines following a hospital visit in 2012. Patients who experienced an improvement of at least 1 modified Rankin Scale (mRS) grade after the intervention were classified as “improved,” while the remaining patients were classified as “non-improved.” In the shunt intervention (*n* = 437) and non-shunt intervention (*n* = 437) groups, the differences in mRS grade improvement were analyzed using the Mann-Whitney *U*-test. Finally, we examined subjects in the shunt intervention group (*n* = 974) to compare the outcomes and complications of ventriculoperitoneal (VP) shunt (n = 417) with lumboperitoneal (LP) shunt (*n* = 540).

**Results:** We examined subjects with iNPH to compare the non-shunt intervention group to the shunt intervention group following adjustment for age and mRS grade at baseline by propensity score matching (0.31–0.901). The mRS grade (mean [SD]) was found to improve with non-shunt intervention (2.46 [0.88]) and shunt intervention (1.93 [0.93]) (*p* < 0.001) in iNPH patients. The mRS outcome score and complications comparison between the VP and LP shunt groups did not show significant difference.

**Conclusions:** In this study, analysis of the efficacy of shunts for possible iNPH conducted in Japan indicated a significant improvement in the mRS grade between baseline and outcome within 1 year, regardless of the surgical technique, and shunt intervention was found to be effective.

## Introduction

Idiopathic normal pressure hydrocephalus (iNPH) is a syndrome that primarily affects elderly individuals and manifests as symptoms such as gait disturbance, cognitive impairment, and urinary incontinence (1). iNPH is characterized by dilation of the cerebral ventricles, normal cerebrospinal fluid (CSF) pressure, and symptomatic improvement after CSF shunting.

The first hospital-based study on iNPH was conducted in 1992 as a multicenter study in the Netherlands by Vanneste et al. and reported an incidence of 2.2 individuals per million persons (2). Thereafter, a study conducted in Norway by Brean et al. reported the prevalence and incidence of suspected cases of iNPH to be 21.9 and 5.5 individuals per 100,000 persons, respectively (3). Lemcke et al. characterized the epidemiology of iNPH based on nationwide insurance claim data in Germany in 2012 and reported an annual incidence of definitive iNPH treated by shunt surgery of 1.08 individuals per 100,000 persons (4). Following improvements in the diagnostic methods for iNPH, a more recent epidemiological investigation of probable iNPH diagnoses by computed tomography (CT) in the general population indicated a prevalence exceeding 5.4% in individuals aged >80 years (5). Thus, it can be inferred that iNPH is not a rare disease in developed countries.

Since 2008, three population-based studies have reported on iNPH in local residents in different regions of Japan (6–8). Other surveys in Japan have examined the prevalence of cases with brain magnetic resonance imaging (MRI) revealing ventricular enlargement with an Evans Index of >0.3 and tightness of high convexity, as well as the prevalence of possible iNPH according to the existing diagnostic and treatment guidelines (9, 10). These reports indicated a prevalence of iNPH of 1.1% (95% confidence interval [CI]: 0.6–1.8%) among local community residents aged ≥60 years (mean age: 70.8 years) in Japan. However, data related to the surgical treatment (CSF shunting) for iNPH and qualitative CSF testing or tap test results (11–13) were not reported in these studies, thus the reported findings may not represent the actual prevalence of iNPH in Japan.

To date, no study has conducted a nationwide epidemiological survey on iNPH in Japan, and only one previous report has described the precise prevalence of iNPH. In the present report, responses were obtained from 1,804 clinical departments (recovery rate: 42.7%). A total of 3,079 patients met the established diagnostic criteria for iNPH (10), and among these, 1,815 underwent shunt surgery. On the basis of these data, it was estimated that 12,900 patients (95% CI: 10,000–15,800) are treated for iNPH in Japan annually, with 6,700 patients (95% CI: 4800–8600) receiving shunt surgery treatment. The estimated prevalence of iNPH in this study was 10.2 individuals per 100,000 persons in 2012 (14).

Such marked discrepancy in epidemiological studies may have been caused by differences in the reported prevalence in the hospital- and regional population-based studies due to general age-related symptoms in elderly people such as gait disturbance and cognitive decline that tend to not be recognized as disease symptoms. We believe that further elucidation is necessary with regard to the diagnosis of this disease and the treatment efficacy.

This study aimed to investigate the efficacy of CSF shunt surgery for iNPH using data from a nationwide epidemiological survey conducted in Japan.

## Methods

### Patients and methods for the nationwide epidemiological survey

The current study consisted of two sequential epidemiological surveys: first, we conducted a primary survey in patients with a diagnosis of iNPH who received medical care during 2012. We then conducted a secondary survey in order to clarify the clinical characteristics and treatment outcomes of these patients. We separately asked attending physicians to confirm specific clinical details via a mailed questionnaire (Supplementary Table 1).

A cross-sectional study was conducted as an extension of a previous nationwide epidemiological survey on iNPH (14). Using this data set, we analyzed the efficacy of surgical treatment for iNPH in Japan.

A nation-wide survey was conducted on patients that (1) were aged ≥60 years; (2) presented with enlargement of the brain ventricles, and (3) presented with one or more of the following symptoms: gait disturbance, cognitive impairment, and urinary incontinence. These patients were considered to be cases of possible iNPH in accordance with the guidelines for the diagnosis and treatment of iNPH in Japan (9, 10).

The departments, lists of medical institutions, and special stratified hospitals were selected for the first and second surveys by the method standardized by the Research Committee on Epidemiology of Intractable Diseases in Japan, following previous nationwide surveys of other diseases (15, 16). Similar to the above reports, survey targets from all iNPH-associated departments in Japan were selected by stratified random sampling.

The departments that were eligible for the survey were randomly extracted (per clinical unit) from a nationwide hospital database after each had been stratified on the basis of the hospital bed capacity as follows: hospitals attached to university schools of medicine (medical universities): 100%, general hospitals with ≥500 beds: 100%; 400–499 beds: 80%; 300–399 beds: 40%; 200–299 beds: 20%, 100–199 beds: 10%; ≤99 beds: 5%; and special hospitals wherein patients are believed to be particularly numerous (special-ranking hospitals): 100%. Extraction was performed using a stratified random sampling method, and the overall extraction rate was approximately 20%. The point estimation of the number of patients in each layer was calculated as follows: estimated number of patients (αi) = the reported number of patients / extraction rate × recovery rate; the point estimation of the total number of patients was calculated as follows: estimated total number of patients = Σαi. The primary survey was conducted in health clinics in each of the following specialities: neurosurgery, neurology, neuropsychiatry, and internal medicine. Finally was estimated the total number of patients in Japan for 2012.

The first survey was conducted in February 2012. The departments included in the survey were requested by mail to report the total number and sex of patients examined for iNPH from January 1 to December 31, 2012. The exact methodology for conducting and completing the surveys was based on previously published reports (15, 16). Reminder letters were mailed to the departments that did not respond to first survey.

**Figure 1:**
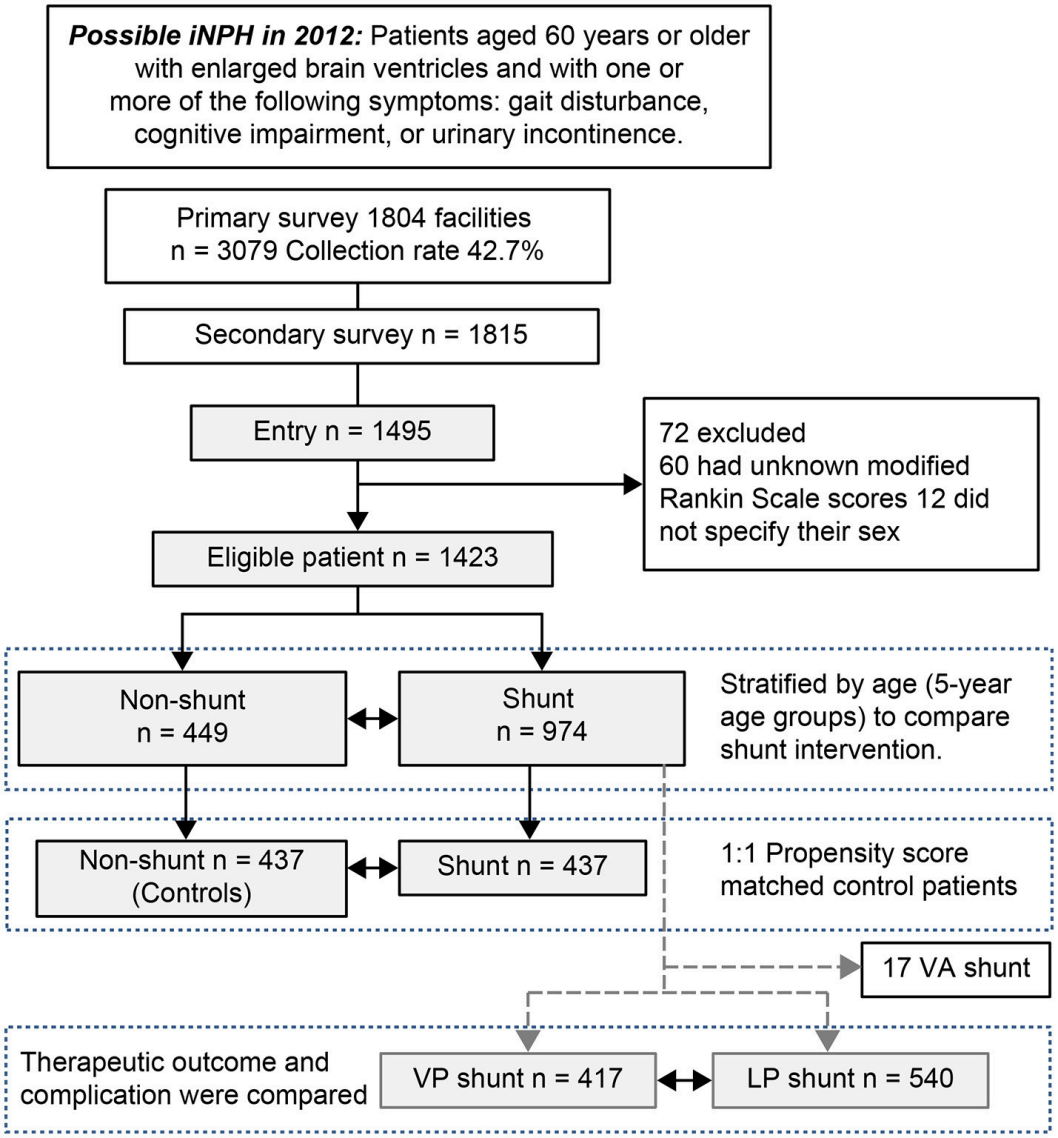
Flow diagram for enrollment of patients with possible idiopathic normal pressure hydrocephalus. Patients with idiopathic normal pressure hydrocephalus (iNPH) who underwent shunt intervention were matched to patients who did not undergo shunt intervention using 1:1 propensity score matching. Confounding factors affecting intervention outcome in the secondary survey were tabulated using cross tabulation and analyzed using a binomial logistic regression analysis and Wald chi-squared test for the propensity score. Finally, we examined subjects in the shunt intervention group to compare the outcomes and complications of ventriculoperitoneal (VP) shunts and lumboperitoneal (LP) shunts. VA, ventriculoatrial.

**Figure 2:**
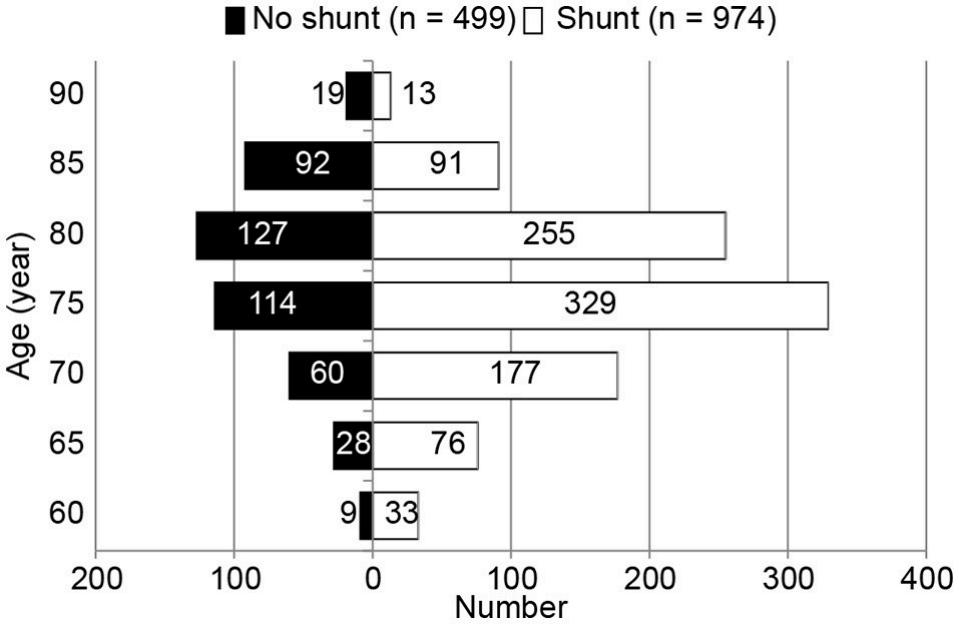
Age-distribution histogram of 1,423 patients with possible idiopathic normal pressure hydrocephalus (iNPH). Data represent the number of possible idiopathic normal pressure hydrocephalus patients (*n* = 1,423). Patients were stratified by age groups (5-year age groups ranging from 60 to 94 years of age) to compare the no shunt group with the shunt group.

Among the 1,495 patients who were enrolled based on the completion of both surveys, 60 had unknown modified Rankin Scale (mRS) scores (17) and 12 did not specify their sex and were excluded from the analysis (flow chart, Figure 1). Thus, a total of 1,423 patients with possible iNPH (842 males and 581 females; mean age [SD]: 76.5 [6.4] years) were included in the analysis (histogram, Figure 2). Propensity score matching (0.31–0.901) was used to select 874 patients (shunt [n = 437] vs. no shunt [*n* = 437]) from the total 1,423 patients (shunt [n = 974] vs. no shunt [*n* = 449]). Patients who experienced an improvement of at least 1 mRS grade after the intervention were classified as “improved,” while the remaining patients were classified as “non-improved” (Table 1).

**Table 1:** Baseline characteristics of 1,432 patients with or without shunt intervention in idiopathic normal pressure hydrocephalus.

	**Unadjusted**			**Matched (1:1)**		
	**Non-shunt [number]**	**Shunt [number]**	***p*-value**	**Non-shunt [number]**	**Shunt [number]**	***p*-value**
possible iNPH patients (%)	449 (31.6%)	974 (68.4%)		437 (50%)	437 (50%)	
Age: mean (SD)	77.9 (6.62)	75.8 (6.12)	< 0.001	77.4 (6.48)	77.0 (5.50)	0.184
Gender: male (%)	264 (58.8%)	578 (59.3%)		257(58.8%)	258 (59%)	
**INITIAL SYMPTOMS**						
Gait disturbance (%*)*	297 (66.1%)	749 (76.9%)		291 (66.5%)	296 (67.7%)	
Cognitive impairment (%)	163 (36.3%)	353 (36.2%)		158 (36.2%)	150 (34.8%)	
Urinary incontinence (%)	65 (14.5%)	185 (19.0%)		64 (14.6%)	56 (12.8%)	
**EXAMINATION**						
PVI (%)	272 (60.6%)	569 (58.4%)		264 (79.4%)	264 (79.4%)	
CIL (%)	42 (9.4%)	94 (9.7%)		41 (9.4%)	40 (9.2%)	
**COMORBIDITY**						
Hypertension (%)	162 (36.1%)	403 (41.4%)		159 (36.4%)	157 (35.9%)	
Hyperlipidemia (%)	52 (11.6%)	139 (14.3%)		52 (11.9%)	47 (10.8%)	
Diabetes mellitus (%)	70 (15.6%)	181 (18.5%)		68 (15.6%)	63 (14.4%)	
Cervical spondylosis (%)	14 (3.1%)	33 (3.4%)		14 (3.2%)	9 (2.1%)	
Lumbar spondylosis (%)	37 (8.2%)	114 (11.7%)		34 (7.8%)	38 (8.7%)	
Alzheimer's disease (%)	93 (20.3%)	108 (11.1%)		87 (19.9%)	75 (17.2%)	
**OUTCOME**						
mRS grade at baseline: mean (SD)	2.55 (0.71)	2.70 (0.77)	< 0.001	2.51 (0.68)	2.53 (0.69)	0.597
mRS outcome grade: mean (SD)	2.51 (0.88)	2.00 (0.93)	< 0.001	2.46 (0.88)	1.93 (0.93)	< 0.001
mRS improved (%)	64 (14.3%)	582 (59.8%)		61 (14.0%)	240 (54.9%)	

*PVI, Periventricular hyperintensity; CIL, Chronic ischemic lesion; mRS, modified Rankin Scale. Data represent the number (percentage) or mean (SD). We examined subjects with idiopathic normal pressure hydrocephalus to compare the non-shunt intervention group to the shunt intervention group using the Mann-Whitney U test. We adjusted for age, gait disturbance, comorbidity of Alzheimer's disease, and mRS grade at baseline by propensity score matching (0.31–0.901), and confirmation was obtained by the Wilcoxon sign-rank test and McNemar test*.

### Analysis of recovered questionnaires

After the primary survey, questionnaires were conducted in all hospitals with reported cases of iNPH. These questionnaires initially examined sex, age, diagnostic classification entry data, initial symptoms, and comorbidities (Supplementary Table 1). Participating physicians were also requested to describe the results of the patients' (1) cranial MRI (i.e., presence/absence of ventricular dilatation, presence/absence of chronic ischemic lesions ≤1.5 cm, presence/absence of white matter lesions directly below the cortex, and periventricular hyperintensity) and (2) spinal cord MRI (i.e., presence/absence of degenerative spondylosis in the cervical and lumbar spine). Lumbar CSF analysis data were also included; the survey assessed whether CSF tap tests and drainage tests had been performed and the outcomes of these tests. Treatment information included the shunting method and system, complications, and outcome. Therapeutic efficacy was evaluated based on both the attending physician's assessment as well as the mRS grade as an indicator of ability to carry out activities of daily living. We changed the recording of the activities of daily living outcomes in the secondary survey card to the following and calculated the mRS grade.

5 “Able to walk normally” = mRS 1

4 “Able to walk alone while still handicapped” or “unable to perform all previous activities but able to take care for him/herself without assistance” = mRS 2

3 “Able to walk only with a cane” or “requires some help, but able to walk without assistance”

= mRS 3

2 Wheelchair-bound = mRS 4.

### Statistical analysis

The shunt intervention (*n* = 974) and non-shunt intervention (*n* = 449) groups were compared using binomial logistic regression analysis to obtain a propensity score for factors other than the change in mRS grade. Confirmation was obtained by the Wilcoxon sign-rank test and McNemar test.

iNPH patients who underwent shunt intervention were matched to patients who did not undergo shunt intervention using 1:1 propensity score matching (18, 19). The matching variables for patients are presented in Table 1. To assess the pre-match imbalance and post-match balance, the standardized differences were estimated for all baseline covariates before and after matching (20). For a given covariate, a standardized difference of <10% indicates a relatively small imbalance (20). To investigate the differences between the shunt intervention and non-shunt intervention groups, the Mann-Whitney *U*-test was used for categorical variables.

Finally, we examined subjects in the shunt intervention group (*n* = 974) to compare the ventriculoperitoneal (VP) shunt (*n* = 417) and lumboperitoneal (LP) shunt (*n* = 540) procedure outcomes using the Mann-Whitney *U*-test (Table 2) and complications using the Pearson chi-squared test (Table 3). The significance level was set at a two-sided *p* = 0.05. All statistical analyses were performed using the IBM SPSS Statistics 18 package (SPSS Inc. Chicago, IL, USA).

**Table 2:** Baseline data for shunt intervention method comparison (ventriculoperitoneal shunt vs. lumboperitoneal shunt).

	**Shunt total number**	**VP shunt number**	**LP shunt number**	**VP vs. LP *p*-value**
Patient number (%)	974	417 (42.8%)	540 (55.4%)	
Age: mean (SD)	77.29 (6.12)	76.9 (6.20)	77.6 (6.08)	0.087
Gender male (%)	578	240 (57.6%)	326 (60.1%)	
**INITIAL SYMPTOMS**				
Gait disturbance	749	324 (77.3%)	416 (77.0%)	
Cognitive impairment	353	151 (36.1%)	194 (35.9%)	
Urinary incontinence	185	74 (17.7%)	111 (20.6%)	
**EXAMINATION**				
PVI	569	263(63.1%)	305(56.5%)	
CIL	94	46(11.0%)	48(8.9%)	
**COMORBIDITY**				
Hypertension	403	166 (39.8%)	230 (42.6%)	
Hyperlipidemia	139	55 (13.2%)	81 (15%)	
Diabetes mellitus	181	77 (18.5%)	100 (18.5%)	
Cervical spondylosis	33	16 (3.8%)	17 (3.1%)	
Lumbar spondylosis	114	58 (13.9%)	50 (9.3%)	
Alzheimer disease	108	38 (9.1%)	67 (12.4%)	
**OUTCOME**				
mRS grade at baseline: mean (SD)	2.70 (0.77)	2.73 (0.76)	2.66 (0.76)	0.561
mRS outcome grade: mean (SD)	2.00 (0.93)	2.01 (0.92)	1.98 (0.93)	0.927
mRS improved	582	254 (60.9%)	317 (58.7%)	

*Data represent the number (percentage) or mean (standard deviation)*.

*VP, ventriculoperitoneal; LP, lumboperitoneal; PVI, Periventricular hyperintensity; CIL, Chronic ischemic lesion; mRS, modified Rankin Scale*.

*We examined subjects in the shunt intervention group to compare ventriculoperitoneal shunts to lumboperitoneal shunts using the Mann-Whitney U-test*.

**Table 3:** Differences in complications due to differences in shunt treatment method (ventriculoperitoneal shunt vs. lumboperitoneal shunt).

**VP shunt (*n* = 417)**	**Number**	**(%)**	**LP shunt (*n* = 540)**	**Number**	**(%)**	***p*-value**
Chronic subdural hematoma effusion (included acute subdural hematoma)	15 (1)	3.5	Chronic subdural hematoma	21	3.8	0.783
Shunt malfunction	11	2.6	Shunt malfunction	19	3.5	0.418
Infection	6	1.4	Hypotension headache	13	2.4	0.164
Hypotension headache	5	1.2	Low back·leg pain	12	2.2	0.302
Intracranial hematoma	2	0.5	Infection	4	0.7	0.445
Subcortical hematoma	1	0.2	Subcortical hematoma	3	0.5	0.862
Cerebral infarction	1	0.2	Cerebral thrombosis	1	0.2	0.862
Pneumothorax	1	0.2	Epigastric hernia	1	0.2	
Epigastric hernia	1	0.2	Silicon allergy	1	0.2	
			Seizure	1	0.2	
			Difficulty elevating the shoulders	1	0.2	
	43	10.0%		77	14.1%	0.074

*iNPH, idiopathic normal pressure hydrocephalus; VP, ventriculoperitoneal; LP, lumboperitoneal. Data represent the number and percentage. Ventriculoperitoneal shunt malfunction: deviation at the abdominal side of the shunt tube; lumboperitoneal shunt malfunction (n = 19): obstruction of the shunt tube at the lumbar spine side (n = 10) and at the abdominal side (n = 8); under-drainage (n = 1). We examined subjects to compare the complications using Pearson chi-square tests*.

### Ethical review

Patient consent was neither required nor sought, as this study was conducted in compliance with the ethical guidelines for epidemiological research (Notification No. 1 by the Ministry of Education, Culture, Sports and the Ministry of Health, Labor and Welfare in 2007). Patients' personal identification data were preserved according to all existing regulations regarding the handling of such data according to the Ministry of Health, Welfare and Labor. This study was approved by the ethical committees of our institute.

## Results

In the first survey, 4,220 of a total of 14,089 hospitals (459 university hospitals, 13,582 general hospitals, and 48 special stratified hospitals) were extracted, and an epidemiological investigation was carried out by mail. In the primary survey, responses were obtained from 1,804 clinical departments (recovery rate: 42.7%). Patients who met the diagnostic criteria for iNPH in accordance with the second edition of the guidelines pertaining to iNPH accounted for 3,079 individuals, and those who underwent shunt surgery as a treatment accounted for 1,815 individuals.

The clinical characteristics of the iNPH patients registered in the second survey were then summarized. In the second survey, valid answers were obtained from 1,495 patients. Of these, due to incomplete data, only 1,423 patients were selected and analyzed.

### Diagnostic approach

The CSF tap test is not listed as a required procedure in the guidelines for diagnosing iNPH in Japan (10); yet, this test was performed in 1,256/1,423 patients (84%), not performed in 173 patients (11.6%), and no result specified in 66 patients (4.4%). The most common reason for not performing the tap test was severe gait disturbance interfering with the patient's ability to perform the 3-min timed “up-and-go” test. The tap test was positive in 1,047 patients (83.3%).

### Therapeutic efficacy

A total of 1,423 patients with possible iNPH were divided into 5-year age categories, and the distribution of shunt (*n* = 974) and non-shunt intervention (*n* = 499) was determined (Figure 2). Analysis of the two groups using the Mann-Whitney *U*-test revealed statistically significant differences in age and mRS grade at baseline (Table 1). The mean age at the time of treatment was significantly lower in the shunt intervention group than in the non-shunt intervention group (OR: 0.960, 95% CI: 0.939–0.980, *p* < 0.001). A greater number of patients experienced gait disturbance in the shunt intervention group than in the non-shunt intervention group (OR: 0.571, 95% CI: 0.415–0.786, *p* = 0.001). Additionally, the mRS grade at baseline was higher in the shunt intervention group than in the non-shunt intervention group (OR: 1.212, 95% CI: 1.012–1.451, *p* = 0.037). Comorbidity of Alzheimer's disease was less frequent in the shunt intervention group than in the non-shunt intervention group (OR: 1.586, 95% CI: 1.109–2.267, *p* = 0.012). Propensity scores were calculated for background factors including the mRS grade at baseline, age at the time of treatment, presence of gait disturbance, and comorbidity of Alzheimer's disease. The propensity score (0.31–0.901) for the two groups was calculated and the two groups were prepared for matching (1:1) (*n* = 437 each).

Shunt intervention for iNPH patients was found to be significantly more effective than non-shunt intervention for improving the mRS grade (mean [SD]): shunt intervention = 2.46 [0.88], non-shunt = 1.93 [0.93] (*p* < 0.001).

### Complications of shunt treatment

**Figure 3:**
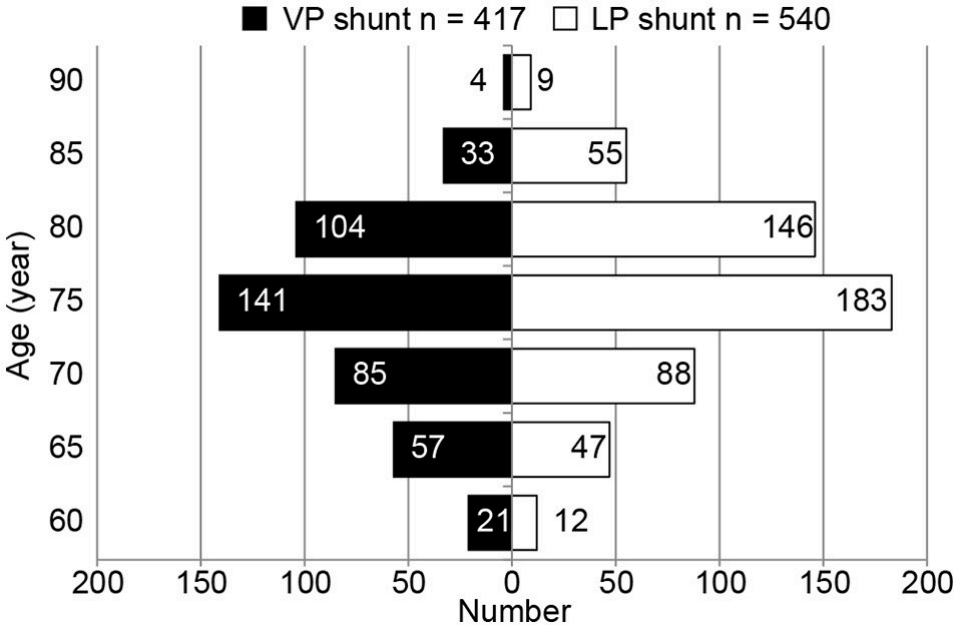
Histogram of shunt intervention (ventriculoperitoneal shunt vs. lumboperitoneal shunt) in idiopathic normal pressure hydrocephalus patients. Data represent the number of patients receiving shunt interventions (ventriculoperitoneal [VP] shunt, *n* = 417; lumboperitoneal [LP] shunt, *n* = 540). Patients were stratified by age (5-year age groups) to compare the two different shunt procedures.

A comparison of patient groups including those who underwent VP shunt or LP shunt, the two most common shunt therapies (histogram, Figure 3), revealed that differences in shunt technique had no effect on patient outcome (Table 2).

Of the 974 patients who received shunt treatment, 120 (12.3%) reported complications: 77 patients (14.1%) and 43 patients (10%) experienced complications after LP and VP shunt, respectively, with no significant difference in the prevalence of complications according to treatment method (*p* = 0.074). In the 77 patients who experienced complications after LP shunt, 19 (3.5%) experienced obstruction of the lumbar catheter, 21 (3.8%) developed a chronic subdural hematoma, 13 (2.4%) experienced symptoms of intracranial hypotension (e.g., headache), and 12 (2.3%) reported lower back or leg pain. In the 43 patients who experienced complications after VP shunt, 15 (3.5%) developed a chronic subdural hematoma and 11 (2.6%) experienced shunt malfunction (Table 3).

## Discussion

The present study is the first to provide an analysis of epidemiological and treatment outcome data for iNPH in a Japanese cohort. Japan is one of the world's most rapidly aging societies, such that the incidence of age-related diseases such as iNPH is predicted to increase in the near future. Our report demonstrated that older patients (particularly those aged >80 years) were less likely to receive shunt treatments. Therefore, accurate diagnosis of iNPH in outpatient populations and subsequent shunt treatment is critical. A main reason for misdiagnosis of iNPH is the misinterpretation of gait and dementia symptoms as manifestations of normal aging in elderly patients. Thus, medical professionals may not suspect iNPH or may hesitate to recommend surgery.

In a prospective study of iNPH in Japan, shunt surgery efficacy was approximately 80% in cases diagnosed with one or more of the three cardinal symptoms present (21). The efficacy was similar when characteristic findings were identified by diagnostic imaging, including ventricular dilatation and narrowing of the subarachnoid space on the internal surface of the cerebral hemispheres and superior fornix or enlargement of the Sylvian fissure. In the SINPHONI study, these imaging findings were collectively termed “disproportionately enlarged subarachnoid-space hydrocephalus” (21, 22). In light of these results, a revised edition of the Japanese guidelines was published in 2011, indicating shunt surgery without a CSF tap test in cases presenting with gait disturbance and clear disproportionately enlarged subarachnoid-space hydrocephalus with ventricular dilatation (10). Although the present study was conducted after the publication of these revised guidelines, we noted that tap tests were performed in >80% of the cases included in the analysis. This may have been due to the fact that, for attending physicians as well as patients and family members, a positive tap test result was considered to be a robust indication for shunt surgery compared to other findings (23, 24).

In this study, the great majority of cases that were treated with either VP shunt or LP shunt. The LP shunt is a minimally invasive approach, which has established its status as the most popular treatment for iNPH management in Japan (25–27). Both VP shunt and LP shunt resulted in mRS grade improvements in patients with probable iNPH, with no significant difference in treatment outcome between the two approaches (28). Although a statistically significant difference was not found, it is presumed that LP shunts are associated with a greater number of surgical complications leading to reoperation.

This study has several limitations. First, this was a survey-based study that may have been subject to recall bias and other forms of bias. Second, we did not assess the time from diagnosis to treatment or its effect on surgical outcome. Third, our study employed a short follow-up period. Fourth, we must also consider the indication bias of shunt intervention. In particular, for older patients and comorbid patients, the surgeon's choice of invasive intervention was likely determined by the expectation of a favorable treatment outcome that would increase the response rate in the treated cohort. Therefore, we adjusted the confounding factors and compared the two groups by 1:1 matching. These limitations should be considered in the design of future studies that will aim to confirm our findings.

## Conclusions

This is the first hospital-based survey study of the efficacy of shunt intervention in Japanese patients with possible iNPH. The analysis of shunt intervention for possible iNPH conducted in Japan indicated a significant improvement in the mRS grade between baseline and outcome within 1 year, regardless of the surgical technique, and shunt intervention was found to be effective.

## Data availability statements

Datasets are available upon request. The raw data supporting the conclusions of this manuscript will be made available by the authors, without undue reservation, to any qualified researcher.

## Author contributions

The study was carried out collaboratively. MN, MM, IO, CA, KK, MK, NK, YW, WF, EM, TK, HS, KK, and HA participated in the conception of the study and development of the study design. MN, MM, NK, MK, WF, YW, EM, and HA collected the data. MN, MM, IO, MK, NK, HS, KK, and HA analyzed the data, interpreted the results, and drafted the manuscript. HA was the principal investigator of this study. MN wrote the original draft of the paper, and all authors contributed to critical revision. All authors have seen and approved the final version to be published.

### Conflict of interest statement

The authors declare that the research was conducted in the absence of any commercial or financial relationships that could be construed as a potential conflict of interest.

## Abbreviations

CI: confidence interval
CSF: cerebrospinal fluid
iNPH: idiopathic normal pressure hydrocephalus
LP: lumboperitoneal
MRI: magnet resonance imaging
mRS: modified Rankin Scale
OR: odds ratio
VP: ventriculoperitoneal.

## References

[1] Adams RD Fisher CM Hakim S Ojemann RG Sweet WH. Symptomatic occult hydrocephalus with “Normal” cerebrospinal-fluid pressure: a treatable syndrome. N Engl J Med. (1965) 273:117–26. 10.1056/nejm196507152730301 14303656

[2] Vanneste J Augustijn P Dirven C Tan WF Goedhart ZD. Shunting normal-pressure hydrocephalus: do the benefits outweigh the risks? A multicenter study and literature review. Neurology (1992) 42:54–9. 10.1212/wnl.42.1.54 1734324

[3] Brean A Eide PK. Prevalence of probable idiopathic normal pressure hydrocephalus in a Norwegian population. Acta Neurol Scand. (2008) 118:48–53. 10.1111/j.1600-0404.2007.00982.x 18205881

[4] Lemcke J Stengel D Stockhammer F Güthoff C Rohde V Meier U Nationwide incidence of Normal Pressure Hydrocephalus (NPH) assessed by insurance claim data in Germany. Open Neurol J. (2016) 26:15–24. 10.2174/1874205x01610010015 PMC4891984 27330575

[5] Jaraj D Rabiei K Marlow T Jensen C Skoog I Wikkelsø C. Prevalence of idiopathic normal-pressure hydrocephalus. Neurology (2014) 82:1449–54. 10.1212/wnl.0000000000000342 24682964 PMC4001197

[6] Iseki C Kawanami T Nagasawa H Wada M Koyama S Kikuchi K. Asymptomatic ventriculomegaly with features of idiopathic normal pressure hydrocephalus on MRI (AVIM) in the elderly: a prospective study in a Japanese population. J Neurol Sci. (2009) 277:54–7. 10.1016/j.jns.2008.10.004 18990411

[7] Hiraoka K Meguro K Mori E. Prevalence of idiopathic normal-pressure hydrocephalus in the elderly population of a Japanese rural community. Neurol Med Chir. (2008) 48:197–200. 10.2176/nmc.48.197 18497491

[8] Tanaka N Yamaguchi S Ishikawa H Ishii H Meguro K. Prevalence of possible idiopathic normal-pressure hydrocephalus in Japan: the Osaki-Tajiri project. Neuroepidemiology (2009) 32:171–5. 10.1159/000186501 19096225

[9] Ishikawa M Hashimoto M Kuwana N Mori E Miyake H Wachi A. Guidelines for management of idiopathic normal pressure hydrocephalus. Neurol Med Chir. (2008) 48:S1–23. 10.2176/nmc.48.s1 18408356

[10] Mori E Ishikawa M Kato T Kazui H Miyake H Miyajima M Guidelines for management of idiopathic normal pressure hydrocephalus. 2nd Edn. Neurol Med Chir. (2012) 52:775–809. 10.2176/nmc.52.775 23183074

[11] Wikkelsø C Andersson H Blomstrand C Lindqvist G. The clinical effect of lumbar puncture in normal pressure hydrocephalus. J Neurol Neurosurg Psychiatry (1982) 45:64–9. 7062072 10.1136/jnnp.45.1.64 PMC491267

[12] Vanneste JA. Diagnosis and management of normal-pressure hydrocephalus. J Neurol. (2000) 247:5–14. 10.1007/s004150050003 10701891

[13] Kahlon B Sundbarg G Rehncrona S. Comparison between the lumbar infusion and CSF tap tests to predict outcome after shunt surgery in suspected normal pressure hydrocephalus. J Neurol Neurosurg Psychiatry (2000) 73:721–6. 10.1136/jnnp.73.6.721 12438477 PMC1757331

[14] Kuriyama N Miyajima M Nakajima M Kurosawa M Fukushima W Watanabe Y. Nationwide hospital-based survey of idiopathic normal pressure hydrocephalus in Japan: epidemiological and clinical characteristics. Brain Behav. (2017) 7:e00635. 10.1002/brb3.635 28293475 PMC5346522

[15] Fukushima W Fujioka M Kubo T Tamakoshi A Nagai M Hirota Y. Nationwide epidemiologic survey of idiopathic osteonecrosis of the femoral head. Clin Orthop Relat Res. (2010) 468:2715–24. 10.1007/s11999-010-1292-x 20224959 PMC2939331

[16] Nakamura Y Matsumoto T Tamakoshi A Kawamura T Seino Y Kasuga M. Prevalence of idiopathic hypoparathyroidism and pseudohypoparathyroidism in Japan. J Epidemiol. (2000) 10:29–33. 10.2188/jea.10.29 10695258

[17] van Swieten JC Koudstaal PJ Visser MC Schouten HJ van Gijn J. Interobserver agreement for the assessment of handicap in stroke patients. Stroke (1988) 19:604–7. 10.1161/01.str.19.5.604 3363593

[18] Randolph JJ Falbe K Manuel AK Balloun JL A step-by-step guide to propensity score matching in R. Pract Assess Res Eval. (2014) 19:1–6. Available online at: http://pareonline.net/getvn.asp?v=19&n=18

[19] Harris H Horst SJ A brief guide to decisions at each step of the propensity score matching process. Pract Assess Res Eval. (2016) 21:1–11. Available online at: http://pareonline.net/getvn.asp?v=21&n=4

[20] Normand ST Landrum MB Guadagnoli E Ayanian JZ Ryan TJ Cleary PD. Validating recommendations for coronary angiography following acute myocardial infarction in the elderly: a matched analysis using propensity scores. J Clin Epidemiol. (2001) 54:387–98. 10.1016/S0895-4356(00)00321-8 11297888

[21] Hashimoto M Ishikawa M Mori E Kuwana N Study of INPH on neurological improvement (SINPHONI). Diagnosis of idiopathic normal pressure hydrocephalus is supported by MRI-based scheme: a prospective cohort study. Cerebrospinal Fluid Res. (2010) 7:18 10.1016/s0303-8467(08)70023-4 21040519 PMC2987762

[22] Akiguchi I Shirakashi Y Budka H Watanabe Y Watanabe T Shiino A. Disproportionate subarachnoid space hydrocephalus-outcome and perivascular space. Ann Clin Transl Neurol. (2014) 1:562–9. 10.1002/acn3.87 25356428 PMC4184559

[23] Ishikawa M Hashimoto M Mori E Kuwana N Kazui H. The value of the cerebrospinal fluid tap test for predicting shunt effectiveness in idiopathic normal pressure hydrocephalus. Fluids Barriers CNS (2012) 9:1. 10.1186/2045-8118-9-1 22239832 PMC3293050

[24] Yamada S Ishikawa M Miyajima M Atsuchi M Kimura T Kazui H. Timed up and go test and shunt surgery in idiopathic normal pressure hydrocephalus. Neurol Clin Pract. (2017) 7:1-11. 10.1212/cpj.0000000000000334 29185546 PMC5669413

[25] Kazui H Miyajima M Mori E Ishikawa M. SINPHONI-2 Investigators. Lumboperitoneal shunt surgery for idiopathic normal pressure (SINPHONI-2): an open-label randomised trial. Lancet Neurol. (2015) 14:585–94. 10.1016/S1474-4422(15)00046-0 25934242

[26] Nakajima M Miyajima M Ogino I Sugano H Akiba C Domon N. Use of external lumbar cerebrospinal fluid drainage and lumboperitoneal shunts with strata NSC valves in idiopathic normal pressure hydrocephalus: a single-center experience. World Neurosurg. (2015) 83:387–93. 10.1016/j.wneu.2014.08.004 25108293

[27] Nakajima M Bando K Miyajima M Arai H. Lumboperitoneal shunt placement using computed tomography and fluoroscopy in conscious patients. J Neurosurg. (2009) 111:618–22. 10.3171/2009.1.jns08204 19249929

[28] Miyajima M Kazui H Mori E Ishikawa M on behalf of the SINPHONI-2 Investigators. One-year outcome in patients with idiopathic normal-pressure hydrocephalus: comparison of lumboperitoneal shunt to ventriculoperitoneal shunt. J Neurosurg. (2016) 125:1483–92. 10.3171/2015.10.jns151894 26871203

